# Double-stage insect recognition model based on synergistic localization of key parts

**DOI:** 10.3389/fpls.2026.1854371

**Published:** 2026-06-08

**Authors:** Dongmei Chen, Yimiao Shi, Shaopeng Zhang, Jun Huang, Jingcheng Zhang

**Affiliations:** 1 School of Artificial Intelligence, Hangzhou Dianzi University, Hangzhou, China; 2 Institute of Plant Protection and Microbiology, Zhejiang Academy of Agricultural Sciences, Hangzhou, China

**Keywords:** adaptive synergistic mechanism, cascaded CNN, deep learning, double-stage recognition, insect classification

## Abstract

**Introduction:**

Currently, pests and diseases have caused great losses in global agricultural production systems. However, the Solenopsis invicta has a high degree of interspecific similarity and often pose a great challenge in the accurate identification of insects.

**Methods:**

In order to distinguish closely related species of small insects, this study proposes a Double-stage Insect Recognition Model (DIRM) based on the synergistic localization of key anatomical sites. The model uses the object detection network to pinpoint and crop the key part regions of the target object, and then feed these regions into the classification network and fuse the centroid information, the Global Context Enhancer (GCE) module, and the adaptive synergistic mechanism to linearly synthesis the features of each part in order to efficiently and accurately identify morphologically similar ant species.

**Results:**

Experimental results demonstrate that this approach substantially improves the classification accuracy of morphologically similar ant species, achieving over 95% accuracy on the constructed Ant7C dataset. Compared with conventional single-stage deep learning classifiers, the proposed DIRM method exhibits greater robustness in handling small targets, complex backgrounds, and closely related species.

**Discussion:**

Beyond its applicability to ant identification, the model holds potential for broader applications in the recognition of insects and ecological monitoring.

## 1 Introduction

According to the Food and Agriculture Organization of the United Nations (FAO), about 20-40% of the world’s annual crop production is destroyed by pests and diseases, with a direct economic loss of more than US$ 220 billion (Oerke, 2006). Invasive insects alone cause at least US$70 billion/year, significantly driving up farm costs and quarantine pressures in addition to weakening food supplies (Bradshaw et al., 2016; Faulkner et al., 2020). International plant health standards under the IPPC/SPS framework inform quarantine measures in global trade (Afriansyah and Ardiastuti, 2017). As a result, focus has begun to be placed on the accurate identification of insect species. Early classification of pests and diseases relied heavily on morphological features such as body shape and color (Dubois, 2010; Audisio, 2017). However, these traditional methods usually require specialized equipment and expert knowledge, resulting in complex, costly and time-consuming processes, thus limiting their large-scale application (Yalcin, 2015; Jiang et al., 2019). In recent years, automatic identification methods, including DNA barcoding, spectral analysis and image-based classification, have advanced as new ways to identify pests (Hebert et al., 2003; Azadbakht et al., 2019; Guan et al., 2022). Barcoding studies have also revealed insect diversity in ecological settings (Liu et al., 2018). Among these, image-based identification techniques have gained widespread attention due to their simplicity, efficiency, and cost-effectiveness, and have increasingly been applied in agricultural pest control and ecological monitoring (Schaffner et al., 2001; Al-Saqer and Hassan, 2011). Beyond local-scale image recognition, large-scale monitoring of pest habitats has also been supported by remote-sensing approaches, such as MODIS-based indicators for locust outbreaks (Liu et al., 2008; Renier et al., 2015). They have also been applied in insect and plankton recognition tasks (Wang et al., 2012; Boniecki et al., 2015; Leow et al., 2015).

With the rapid development of deep learning technology, methods based on deep learning have been extensively applied in agriculture and forestry pest research (Marsh et al., 2013; Schneider et al., 2019; DiLorenzo et al., 2021). In addition, multi-modal fusion strategies have been introduced for plant disease recognition tasks (Zhang et al., 2024). Cross-level fusion strategies have boosted fine-grained agricultural recognition (Kong et al., 2021). Wu et al. (2019) developed the IP102 dataset comprising 102 categories and 75,000 images, providing valuable resources for pest research (Wu et al., 2019). Additionally, Wang et al. (2025) proposed the Insect Mamba model based on SSM technology, capturing comprehensive visual features for pest classification for the first time (Wang et al., 2025). Nonetheless, existing deep learning approaches predominantly extract and identify the overall image features, making them inefficient in capturing the subtle local differences necessary for precise classification of closely related insect species (Amarathunga et al., 2022). Accurate identification remains challenging for insect species with extremely similar appearances. Such closely related species often exhibit only subtle differences in localized anatomical features, which current image recognition techniques primarily focusing on overall morphology struggle to effectively capture and differentiate (Park et al., 2020). Although DNA barcoding and spectral analysis offer precise classification, their high costs and complex procedures restrict their feasibility for real-time, large-scale detection (Audisio, 2017).

Among the invasive species detrimental to agriculture, the Solenopsis invicta is a key research subject due to its high similarity to closely related species. This extremely harmful invasive species is highly reproductive and aggressive, poses a serious threat to agricultural production, public safety and the ecosystem. The toxic proteins in its venom can cause severe allergic reactions and even endanger lives (Hastings et al., 2004; Xu et al., 2012). Areawide suppression programs for fire ants in pastures have been reported (Pereira, 2003). Red fire ants are extremely similar to many other ant species (e.g. the Solenopsis geminata) in terms of their morphological features and are difficult to be distinguished by non-specialists or even trained personnel with the naked eye (Callcott and Collins, 1996; Ascunce et al., 2011). Accurate and efficient identification of such morphologically similar invasive species is therefore essential for effective management and control (Sanchez-Peña et al., 2009).

To address these challenges, Chen et al. (2021) utilized a hierarchical weighted activation map approach to accurately locate fruit flies among 13 fly species (Chen et al., 2021). Similarly, Amarathunga et al. (2022) employed a Vision Transformer (ViT) with a visual attention mechanism for the fine-grained classification of western flower thrips (Frankliniella occidentalis) and onion thrips (Thrips tabaci). However, such methods significantly increase model complexity and parameters, making it difficult to extract features from small targets in practical scenarios. Therefore, we propose a Double-stage Insect Recognition Model (DIRM). The proposed DIRM first precisely locate and magnify key anatomical features, followed by a multi-scale feature extraction framework that captures more effective visual details. Ultimately, the model provides a complete image input to recognition output. This approach is particularly suitable for the invasive alien species management and offers a novel perspective for the advancement of intelligent insect recognition through deep integration of insect recognition tasks. The main contributions of this study are as follows:

1. In response to the challenge of low accuracy in distinguishing morphologically similar ant species using traditional classification methods, this study proposes DIRM. The model employs a detection model to locate the key anatomical regions of the target object and crop them into sub-images. After that these sub-images and the overall image will be passed into the classification network where the features extracted from these images will be analyzed.
2. In the classification stage, we utilize the location information provided by the detection model to segment different parts of ants and input the cropped images into the classification network. We locate the entire body parts and combine the central point location information of the ant’s head, thorax, and abdomen with the classification network’s output through feature splicing. Finally, using the Global Context Enhancer (GCE), the model dynamically adjusts and integrates global context information, enabling it to better capture the detailed characteristics of ants in complex backgrounds, thereby enhancing classification robustness.
3. To enhance the overall classification performance, this study introduced a adaptive synergistic mechanism. In this mechanism, the classification information and accuracy of various components of the classification model are comprehensively evaluated to obtain the overall classification results. Through this synergistic calculation, the model ultimately outputs the overall classification result to ensure the comprehensive and accurate classification results and generates structural feature maps and classification information for ants.

## 2 Materials and methods

### 2.1 Experimental materials

Currently, the monitoring and identification of red imported fire ants still rely largely on manual surveys. This process is inefficient and usually requires specialized monitoring sites and complicated field procedures. Since ant species differ in body size and anatomical structure (Manting; Lavine et al., 2010), accurate identification of their key morphological features is important for improving monitoring efficiency and supporting effective prevention and control strategies.

**Figure 1:**
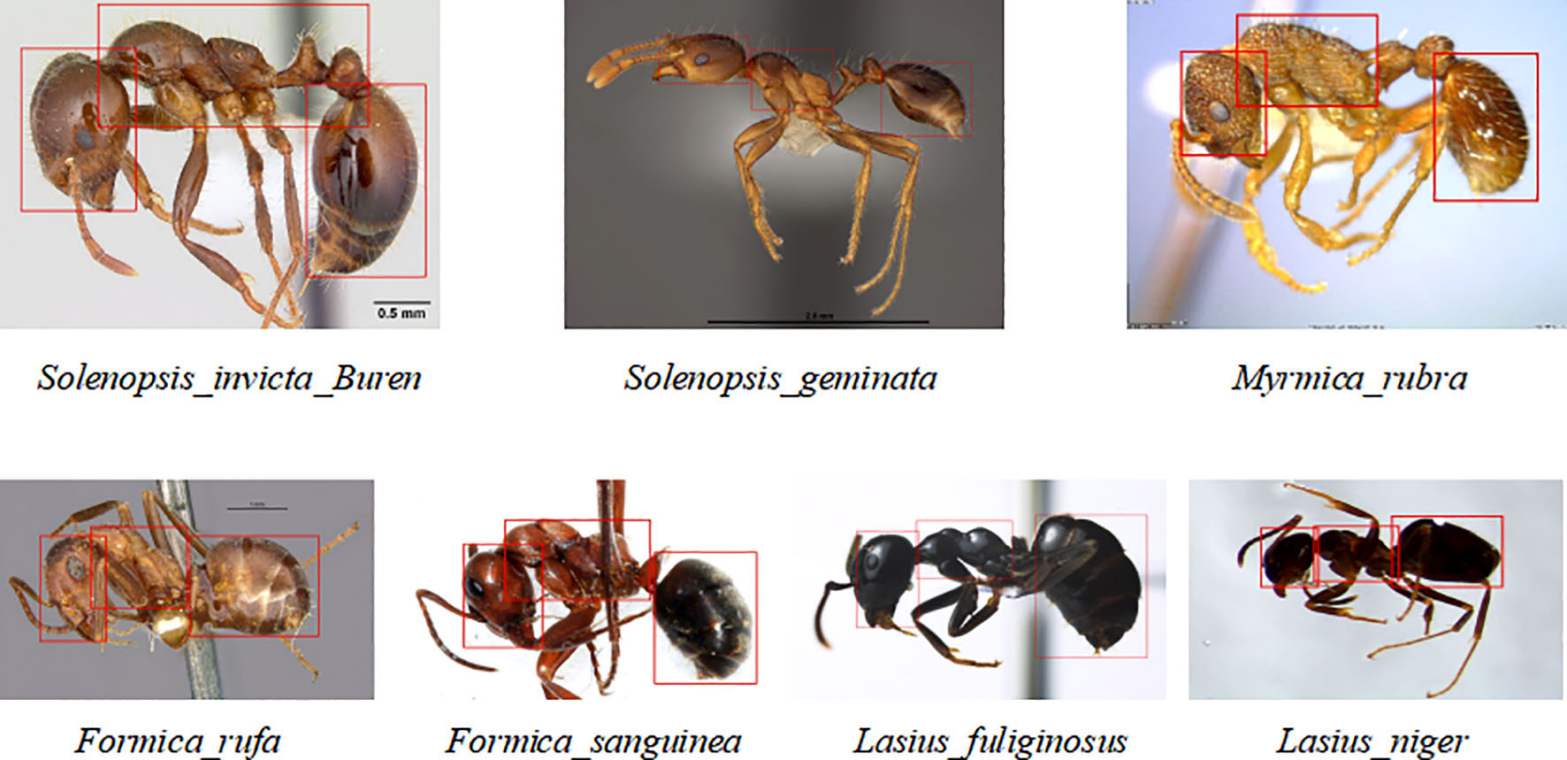
Images of the Ant7C dataset.

To address the challenge of distinguishing morphologically similar ant species, we constructed the Ant7C dataset with the guidance of insect-classification experts. Six common ant species in the family Formicidae were selected because they are morphologically similar to Solenopsis invicta and may be confused with it in field ecosystems. Together with Solenopsis invicta, the dataset contains 3,458 images from seven categories: Solenopsis invicta Buren (538 images), Solenopsis geminata (252 images), Myrmica rubra (594 images), Formica rufa (476 images), Formica sanguinea (455 images), Lasius fuliginosus (956 images), and Lasius niger (187 images) shown in **Figure 1**. The dataset includes single-ant images captured under controlled or semi-controlled specimen-imaging conditions, as well as field images with more complex natural backgrounds. Among these species, Solenopsis invicta and Solenopsis geminata, as well as Lasius fuliginosus and Lasius niger, show highly similar morphological characteristics and are often difficult to distinguish with the naked eye (Kennedy, 2002; Ronque et al., 2016; Ujiyama and Tsuji, 2018; Garikipati and Bond, 2021).

Each image was annotated with three key anatomical regions, the head, thorax, and abdomen, as shown in **Figure 1**. The dataset was divided into training, validation, and test sets at a ratio of 7:2:1. To reduce the risk of data leakage, all derived samples generated from the same source image were kept in the same subset. In addition, the dataset was manually curated to reduce the possibility that visually near-duplicate images of the same individual appeared in different subsets. The anatomical annotations followed a consistent three-part protocol. The tight bounding boxes around the visible head, thorax, and abdomen regions are labeled while excluding legs, antennae, shadows, and irrelevant background whenever possible. Each image was first labeled by a trained annotator and then checked by the research team together with insect experts. Ambiguous cases, inaccurate bounding boxes, and images in which key anatomical structures were severely occluded were corrected or excluded before model training.

### 2.2 Double-stage insect recognition model (DIRM) method

Traditional CNN-based insect classification methods usually use a single-stage network to extract overall features from the whole image and directly classify it (Pattnaik et al., 2020; Du et al., 2022). Hybrid CNN–Transformer frameworks have shown advantages in pest classification (Peng and Wang, 2022). However, this holistic approach is often difficult to access local subtle differences when distinguishing insect species with highly similar appearance. Compared with the conventional one stage end-to-end classification, the proposed method first locates and crops key anatomical parts (head, thorax, abdomen) via a detection module, enabling the model to focus on subtle local morphological differences closely related insect species, rather than relying only on holistic features. It aligns with entomological morphological identification logic, providing better interpretability and practicality for real world insect monitoring and quarantine tasks.

**Figure 2:**
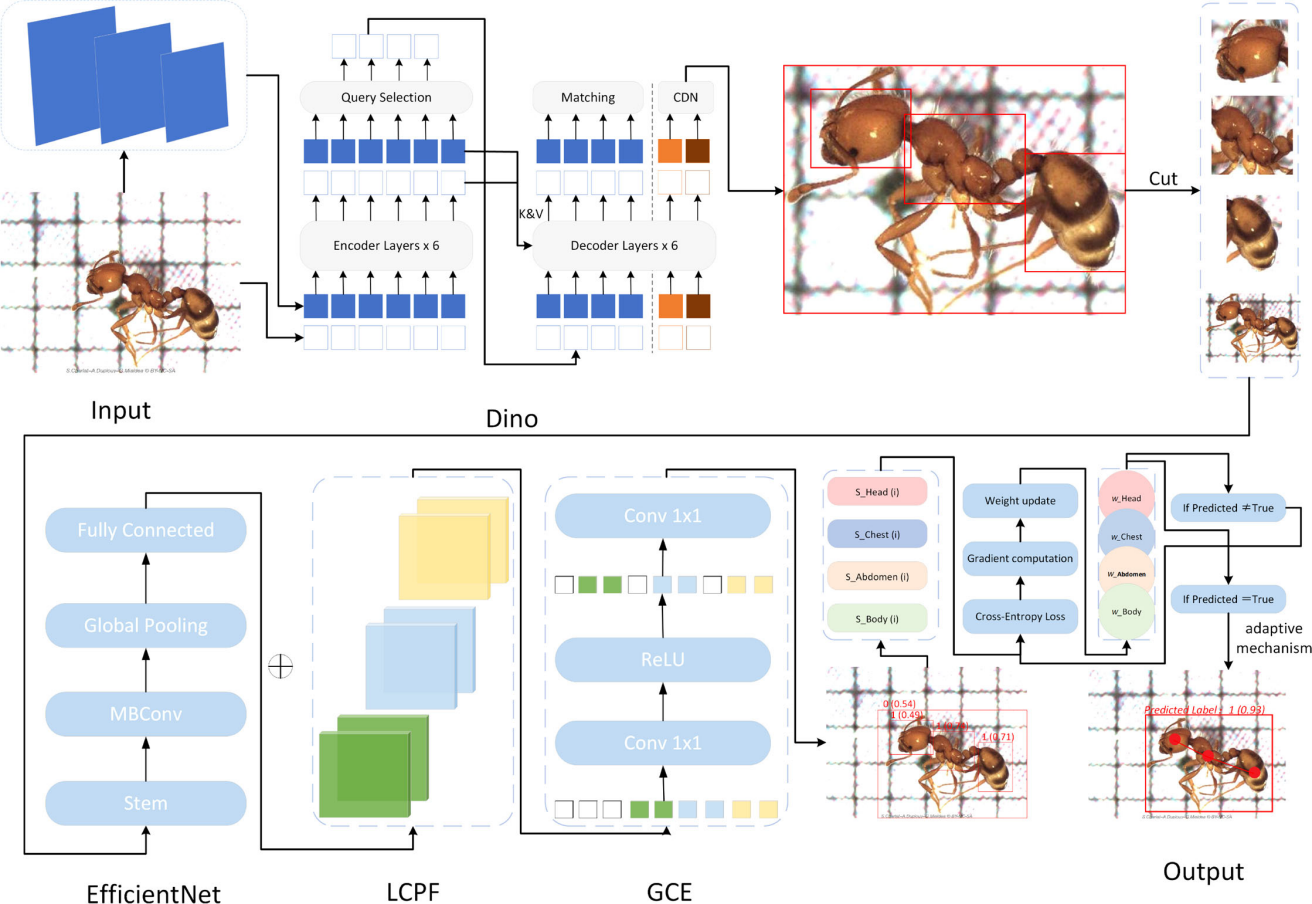
Schematic diagram of the DIRM architecture.

The overall architecture of DIRM based on key part detection, is shown in **Figure 2**. The model applies the DINO detector module. The model first inputs a complete image, then divides it into several fixed-size patches and maps each patch into an embedding vector. Then all the patch embeddings are fed into a six-layer self-attentive Encoder, which integrates the global context information in the layer-by-layer interactions and outputs a set of context-sensitive feature representations. The model prepares a set of learnable queries, which are passed through the six layers of Decoder, and each layer interacts with the information provided by Encoder through a cross-attention mechanism, so as to make the step-by-step predictions of potential detection box locations and category features. The Decoder then outputs candidate frames that correspond to labeled frames through the Matching algorithm, and these queries vectors are mapped to the final border coordinates and category confidence by Component Detection Network (CDN).

After the detection module predicted the bounding boxes for the whole body, head, thorax, and abdomen, each detected region was clipped to the image boundary and cropped from the original RGB image. Before classification, a preprocessing enhancement step was applied to the cropped regions. Specifically, the whole-body bounding box was used to localize the ant body and suppress irrelevant background regions, while denoising was performed on the cropped anatomical regions to reduce noise introduced during detection and cropping. The enhanced crops were then resized to 224×224 pixels and normalized using the ImageNet mean and standard deviation. Therefore, the input tensor of each branch was B×3×224×224. EfficientNet-B0 was used as the shared visual backbone for the four branches, rather than using four independent backbones. For a 224×224 input crop, the final convolutional feature map of EfficientNet-B0 was B×1280×7×7. Global average pooling converted this feature map into a 1280-dimensional visual descriptor, and the final classification layer mapped the descriptor from 1280 dimensions to the seven ant categories.

On the original image, according to the above detection frame, the “whole ant” and “head”, “thorax”, “abdomen” are cropped out and fed into the EfficientNet-B0 backbone network successively. The primary features are extracted by the Stem layer, then refined by the MBConv module for several times, and output by the Global Pooling and Fully Connected Layer and spliced with the centroid position information of the ant’s head, thorax, and abdomen. Further feature processing through the Location-aware Component Position Fusion (LCPF) module generates enhanced feature vectors that jointly encode spatial location information and image features. LCPF uses the detected part-center information as auxiliary positional cues and integrates it with the local appearance descriptors, generating enhanced representations that preserve both morphological details and part-location relationships. This process enhances the model’s ability to understand the local information. Here they are fused together to take into account the details and overall structure of the ant site. The fused feature maps are then entered into the Global Context Enhancer (GCE) module: they are first downscaled by a 1×1 convolution, then activated by ReLU, and finally a channel attention mask is generated using a 1×1 convolution to filter out the noise and highlight the important context. The context-enhanced feature maps are subjected to element-by-element addition operation with the input feature maps, which improves the model’s ability to understand and cope with the overall scene. Then four segmentation heads are fed in parallel: S_head, S_thorax, S_abdomen, and S_body, which output confidence maps of the corresponding parts respectively. Then the cross-entropy loss is calculated for the output of each segmentation head with the real labeling. All the losses are aggregated and backpropagated to update the weights of the backbone network with each segmentation head. Finally, the classification results of each part are integrated and calculated through the adaptive mechanism to generate the final overall classification output.

For spatial encoding, the centroid of each detected part was calculated from its bounding box, and then normalized by the width and height of the original image. For each detected component, the normalized centroid coordinate was used to encode its relative position in the original image. The LCPF module treats this coordinate as a positional prior and fuses it with the corresponding 1280-dimensional EfficientNet-B0 descriptor to obtain a location-aware component representation. In this way, the classifier can exploit not only the visual texture and shape of each cropped part, but also its relative anatomical arrangement within the whole ant body. The GCE module operated on the EfficientNet-B0 feature maps: a 1×1 convolution first aligned the local feature map with the global contextual feature map, ReLU activation was then applied, and a second 1×1 convolution updated the context-enhanced representation. The enhanced feature map was added element-wise to the original feature map before global pooling and classification. Finally, the four branch-level class-score vectors from the head, thorax, abdomen, and whole-body branches were fused at the decision level. The adaptive synergistic mechanism initialized the branch weights equally and updated them according to the validation loss, producing the final class score by a weighted sum of the four branch scores.

Through the synergy of the above detection, classification, the location-aware component position module, and global context enhancement module, the model is able to identify and classify each key part of ants more accurately, providing a more precise and robust solution for intra-class similar pest classification in complex scenarios.

#### 2.2.1 Adaptive synergistic mechanism

We first employ a cascade model to obtain class information and corresponding classification scores for the ant’s head, thorax, abdomen, and overall body. Subsequently, the initial weights are randomly assigned to each component, and a comprehensive score for each class is calculated. Specifically, to determine the overall score ${\text{S}}_{\text{c}}$ for a class ( c), the formula Equation 1 is as follows:

(1) $${\text{S}}_{\text{c}}=\sum_{\text{i}=1}^{\text{n}}{\text{w}}_{\text{i}}\cdot {\text{s}}_{\text{i},\text{c}}$$

where $s_{i,c}$ denotes the score of the i part for class( c), and $w_{i}$ denotes the weight of the i part. After calculating the combined scores for all classes, the class with the highest score is selected as the final predicted class. Subsequently, the weights are adjusted by the gradient backpropagation Equation 2 as follows:

(2) $$\frac{\partial \text{Loss}}{\partial {\text{w}}_{\text{i}}}=\sum_{\text{c}=1}^{\text{C}}({\hat{\text{p}}}_{\text{c}}-{\text{y}}_{\text{c}})\cdot {\text{s}}_{\text{i},\text{c}}$$

where ${\hat{p}}_{c}$ represents the score of the predicted class( c) at position ( i), and $y_{c}$ represents the score of the true class( c) at position( i). The loss value is calculated using the cross-entropy loss function, defined Equation 3 as follows:

(3) $$\text{Loss}=-\sum_{\text{c}=1}^{\text{C}}{\text{y}}_{\text{c}}\cdot \text{log}({\hat{\text{p}}}_{\text{c}})$$

where C is the number of classes, $y_{c}$ is the indicator of the true class (if the class( c) is the true class, then $y_{c}=1$; otherwise, $y_{c}=0$), ${\hat{p}}_{c}$ is the probability of the model’s prediction class( c).

Based on the above three metrics, we incorporated an adaptive synergistic mechanism to improve the efficiency of the contribution of the classification scores of different sites to the final prediction results. In the initial state, the weight of each site is initialized to the same value. During the prediction process, the model dynamically calculates the composite score of each category based on the classification score and classification confidence of each part, and selects the category with the highest composite score as the predicted category.

In the actual prediction, if the predicted class of the model do not differ from the real class, the contribution of each part to the misclassification is calculated by calculating the cross-entropy loss function inversely, and the weight of each part is adjusted adaptively accordingly. If the contribution of a part to misclassification is larger, and the corresponding gradient score is higher, its corresponding weight will be reduced by a larger magnitude, thus reducing the impact of the part in the subsequent prediction; while the weight of the part with a smaller contribution will change less or even remain stable. This dynamic adjustment mechanism enables the model to gradually optimize the contribution weights of different parts to the comprehensive prediction, thus gradually improving the prediction accuracy.

Finally, the weights are updated using gradient descent, and the aforementioned steps are repeated until the model’s predicted category matches the true label. If the labels of all parts do not correspond to the true labels, a classification error is reported.

#### 2.2.2 Metrics

By introducing three metrics including average detection accuracy, classification accuracy, and overall classification accuracy, the model achieves a comprehensive performance evaluation in classification tasks. The average detection accuracy measured the model’s localization accuracy for ant site information, the classification accuracy assessed the model’s prediction accuracy for each site, and the overall classification accuracy reflected the model’s comprehensive performance across all global samples. By integrating these three metrics, the model can more accurately and comprehensively reflect its actual performance in classification tasks, thereby providing a solid foundation for further optimization and improvement.

The mean detection accuracy Det_mAP represents the average accuracy of the model across all classes, Equation 4 as follows:

(4) $$\text{Det}\_\text{mAP}=\frac{1}{\text{C}}\sum_{\text{i}=0}^{\text{C}}{\text{AP}}_{\text{i}}$$

where C is the number of classes and ${\text{AP}}_{\text{i}}$ is the average precision of class i.

Partial classification accuracy ( Cls_pACC) Represents the classification accuracy of the model across all samples. The Equation 5 is as follow:

(5) $$\text{Cls}\_\text{pACC}=\frac{1}{\text{N}}\sum_{\text{i}=0}^{\text{N}}1({\text{y}}_{\text{i}}={\hat{\text{y}}}_{\text{i}})$$

where N is the number of samples, $y_{i}$ is the true label of the i sample, ${\hat{y}}_{i}$ is the prediction label of the i sample, and 1 is the indicator function, which takes the value 1 when the prediction label matches the true label, and 0 otherwise. Overall classification accuracy ( Cls_oACC) is used to evaluate the overall classification performance of the model, representing the classification accuracy of the model across the entire ant sample.

In the model training, computer configuration comprised an Intel(R) Xeon(R) Platinum 8352V CPU @ 2.10GHz, 64GB of RAM, an NVIDIA GeForce RTX 4090 GPU with 24GB of memory, and the Linux Ubuntu 22.04 operating system. In the training phase, the learning rate is 0.001, the learning rate momentum is 0.937, the learning rate decline function is the cosine function, the model optimizer is Adam (adaptive time estimation), the optimizer weight decay factor is 0.0005, the batch size is 4, and the epoch is 50.

The training settings were kept consistent across the ablation experiments. The classification network was initialized with ImageNet-pretrained EfficientNet-B0 weights, trained with cross-entropy loss, and optimized using Adam with an initial learning rate of 0.001, weight decay of 0.0005, batch size of 4, and 50 epochs. The learning rate followed a cosine decay schedule. These settings ensure that the reported gains come from the added spatial encoding, GCE-based context enhancement, and adaptive score fusion rather than from changes in the training protocol.

## 3 Experiment and discussion

### 3.1 Model comparison

In this experiment, we first evaluated the standalone detection networks on the Ant7C dataset. The head, thorax, and abdomen regions were used as the detection targets, and the results are shown in **Table 1**. Among the compared detection models, DINO achieved the highest mAP50 of 94.70%, indicating its stronger localization ability for small anatomical regions.

**Table 1:** Detection results of the mainstream models.

Model	mAP_50	Loss_cls	Loss_bbox	Loss_total
Faster rcnn	93.40%	0.009	0.202	0.211
Fcos	90.80%	0.111	0.220	0.331
Dino	94.70%	0.037	0.177	0.214
Deformable detr	91.30%	0.050	0.213	0.263
Atss	92.00%	0.105	0.298	0.403
DiffusionDet	93.90%	0.004	0.027	0.031
EfficientDet	94.00%	0.298	0.254	0.552

We then evaluated the full-image standalone classification models under the same validation protocol, including both conventional CNN classifiers and the stronger fine-grained baselines with larger backbones or attention mechanisms, as shown in **Table 2**. Among the full-image standalone baselines, EfficientNet-B7 achieved the best validation accuracy of 77.85%, followed by Swin-T at 77.67%, EfficientNet-B4 at 77.52%, and ViT-B/16 at 75.55%. These results show that larger CNN backbones and attention-based architectures substantially improve over the conventional standalone classifiers, and provide stronger baselines for evaluating whether the proposed improvement comes from key-part localization and synergistic decision fusion rather than merely from model capacity.

**Table 2:** Classification results of the mainstream models.

Model	Train-acc	Val-acc	Loss
Mobilenetv2	78.00%	68.00%	0.058
Alexnet	91.10%	67.60%	0.070
convnext	74.40%	63.90%	0.065
efficientnet	79.40%	70.00%	0.055
Resnet101	87.60%	71.70%	0.060
shufflenetv2	67.60%	62.30%	0.068
squeezenet	69.60%	62.50%	0.069
Vgg16	95.50%	67.30%	0.070
ViT-B/16	94.43%	75.55%	0.553
Swin-T	95.49%	77.67%	0.477
EfficientNet-B4	93.75%	77.52%	0.409
EfficientNet-B7	96.51%	77.85%	0.472

**Figure 3:**
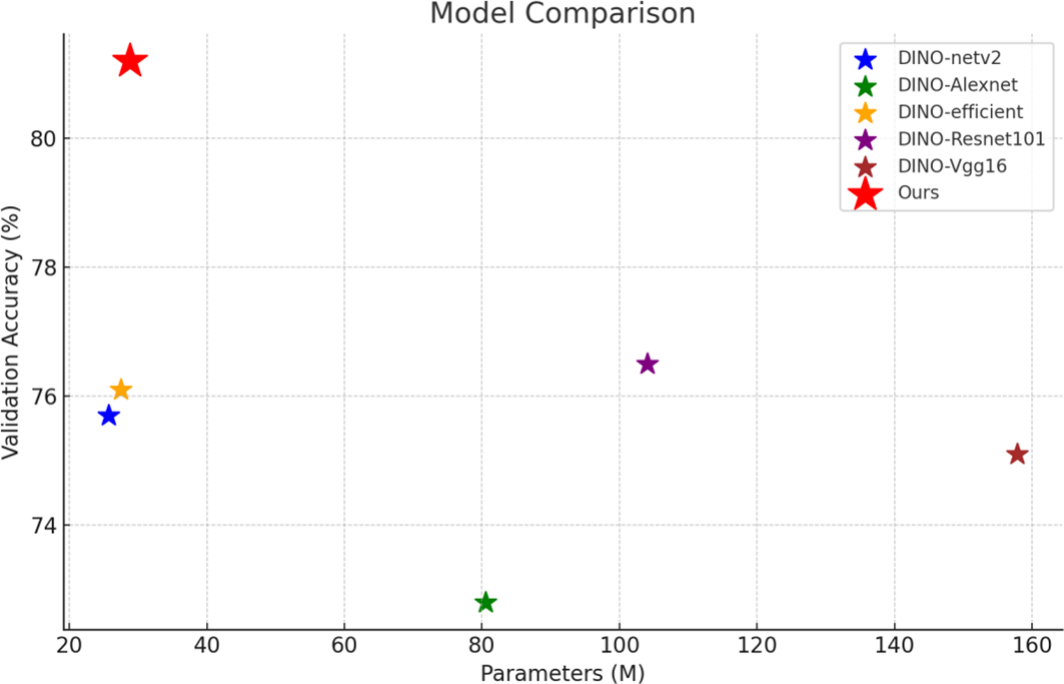
Accuracy comparison of DIRM.

Based on the above results, DINO was used to crop the detected anatomical regions, which were then fed into different classification networks to form DINO-based cascade baselines. As shown in **Figure 3**, the cascade setting allows the classifier to focus on discriminative local regions rather than the entire image. Although DINO-ResNet101 achieved competitive performance among the cascade baselines, DINO-EfficientNet-B0 was selected as the base cascade model considering the balance between classification accuracy, model complexity, and computational efficiency. This base cascade model was then used for the subsequent component analysis and optimization in the ablation study.

### 3.2 Ablation studies

To evaluate the contribution of each component in DIRM, ablation experiments were conducted on the Ant7C validation set using the same data split and evaluation protocol. As shown in **Table 3**, the DINO-EfficientNet-B0 cascade was used as the baseline and achieved a validation accuracy of 81.20% with the preprocessing enhancement.

**Table 3:** Effectiveness comparison of functional modules.

Global context enhancer	Location-aware component position fusion	Adaptive synergistic mechanism			Val-acc
		Equal Weights	Max	Adaptive Mechanism	
					81.20%
**+**					83.87%
		**+**			87.11%
			**+**		90.54%
				**+**	90.86%
**+**	**+**	**+**			93.02%
**+**	**+**		**+**		95.28%
**+**	**+**			**+**	95.48%

After introducing the Global Context Enhancer, the accuracy increased to 83.87%, indicating that contextual feature enhancement helps the model reduce background interference and obtain more discriminative part-level representations. The decision-level fusion strategies further improved the recognition performance. Based on the predictions from different anatomical branches, equal-weight fusion, maximum-selection fusion, and adaptive fusion achieved 87.11%, 90.54%, and 90.86%, respectively. These results show that integrating the branch outputs is more effective than relying on a single cascade prediction. When location-aware component position fusion and GCE were combined with the decision-level strategies, the accuracy increased to 93.02% for equal-weight fusion and 95.28% for maximum-selection fusion. The complete DIRM, which integrates location-aware component position fusion, GCE, and the adaptive synergistic mechanism, achieved the best accuracy of 95.48%. Compared with the 81.20% baseline, the complete model improved accuracy by 14.28 percentage points, demonstrating the effectiveness of progressive feature enhancement and adaptive multi-part decision fusion.

### 3.3 Comparison of adaptive synergistic mechanism

Although the above model can improve the classification accuracy, in the actual detection scene, there will still be inconsistent classification results of different parts of the same image. One possibility is the variability of point site features: the existence of differences in the features of different sites may cause the classification model to produce different classification results on the same image. And another possibility is background information interference: complex background may affect the stability of classification results (Rowe et al., 2024). We analyzed the images with classification errors and found that after introducing the adaptive synergistic mechanism, the model was able to better integrate the information of each part, reducing the classification errors and improving the overall classification accuracy.

**Figure 4:**
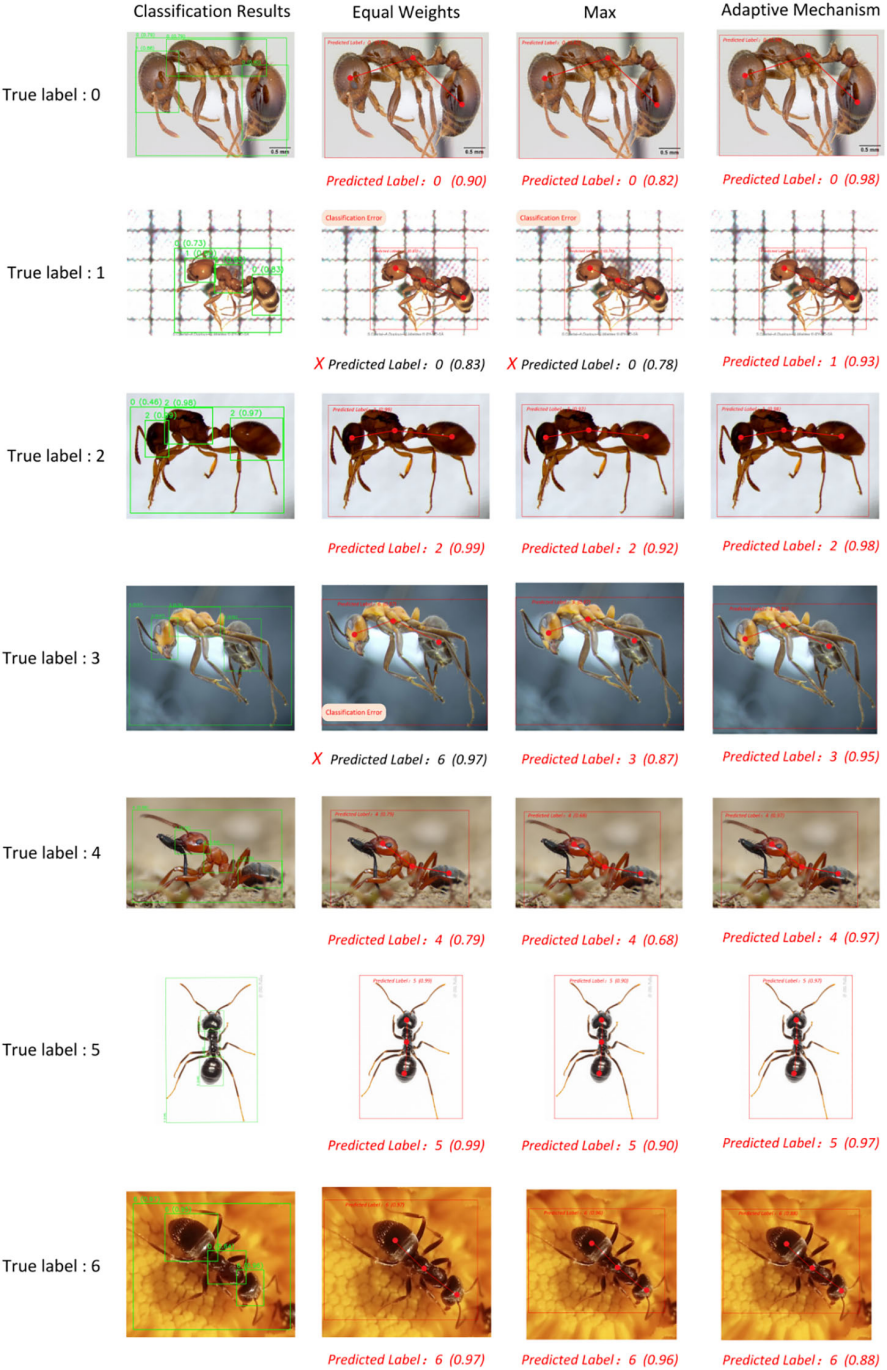
Comparison results of adaptive synergistic mechanism.

As shown in the **Figure 4**, the first column shows the output results of the cascade model, the second column shows the results of adopting the weight equality strategy in the adaptive synergistic mechanism, the third column shows the output results of the maximum selection strategy, and the fourth column shows the output results of the weight adaptive mechanism. It can be seen that in the adaptive synergistic mechanism, the sample with the true label 1 is incorrectly identified as class 0 (the second row and second column in the **Figure 4**), and the sample with the true label 3 is incorrectly identified as class 6 (the second row and second column in the **Figure 4**). In the maximum selection mechanism, samples with a true label of 1 are also incorrectly identified as class 0 (row 2, column 3). However, these errors are effectively corrected in the adaptive synergistic mechanism.

In the adaptive synergistic mechanism, the average score of each category in each part is calculated and the category with the highest score is selected as the final classification result. In the maximum selection mechanism, the maximum of the classification score of each part is directly selected as the final prediction result. The adaptive synergistic mechanism is more flexible and accurate. The weight of each part is dynamically adjusted by the optimization algorithm, and the adaptive adjustment is made according to its influence on the final classification result. Specifically, the model iteratively updates the weights of each part during the training process, so that the weight of the key parts is higher, so that the importance of these parts can be reflected in the classification decision to a greater extent. This adaptive synergistic mechanism not only effectively improves the accuracy of classification, but also enhances the robustness of the model in complex scenarios, and significantly reduces the case of misclassification.

### 3.4 Visual interpretation of different modules

**Figure 5:**
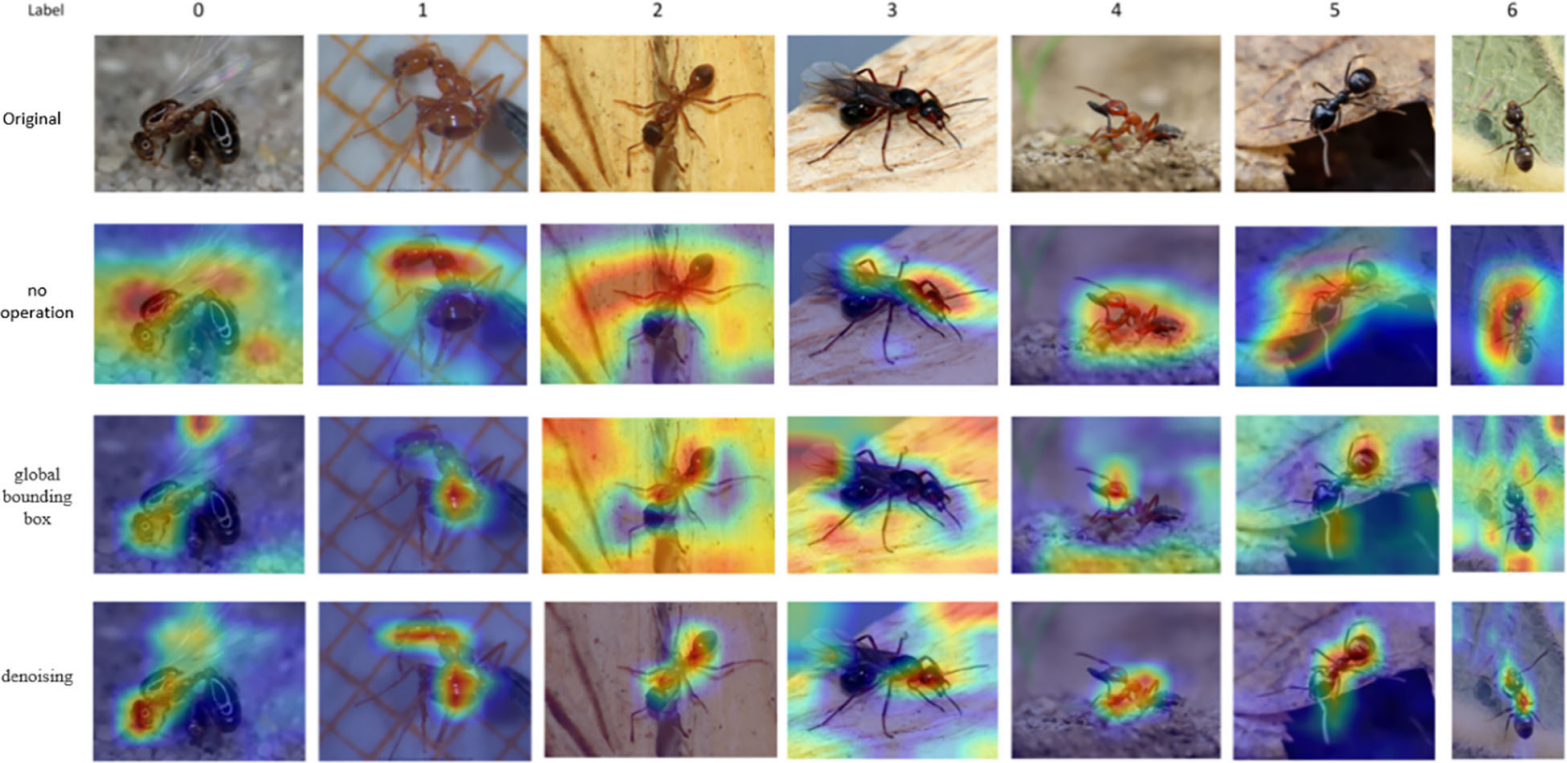
Heat map visualization of different operations.

To visualize the preprocessing enhancement, we applied whole-body localization and denoising before part-level feature extraction. The whole-body bounding box suppresses irrelevant background regions, while denoising reduces noise introduced during detection and cropping. This stage corresponds to the 81.20% accuracy in the ablation study, indicating that cleaner anatomical-region inputs improve the robustness of fine-grained ant classification. To better demonstrate the effectiveness of these improvements, we conducted a visual analysis, as shown in **Figure 5**. The first row displays seven ant categories, the second row shows the original images, the third row presents heatmap visualizations after applying the whole-body bounding box strategy. The results clearly indicate that these strategies help the model focus more precisely on the ant itself, reducing background interference.

**Figure 6:**
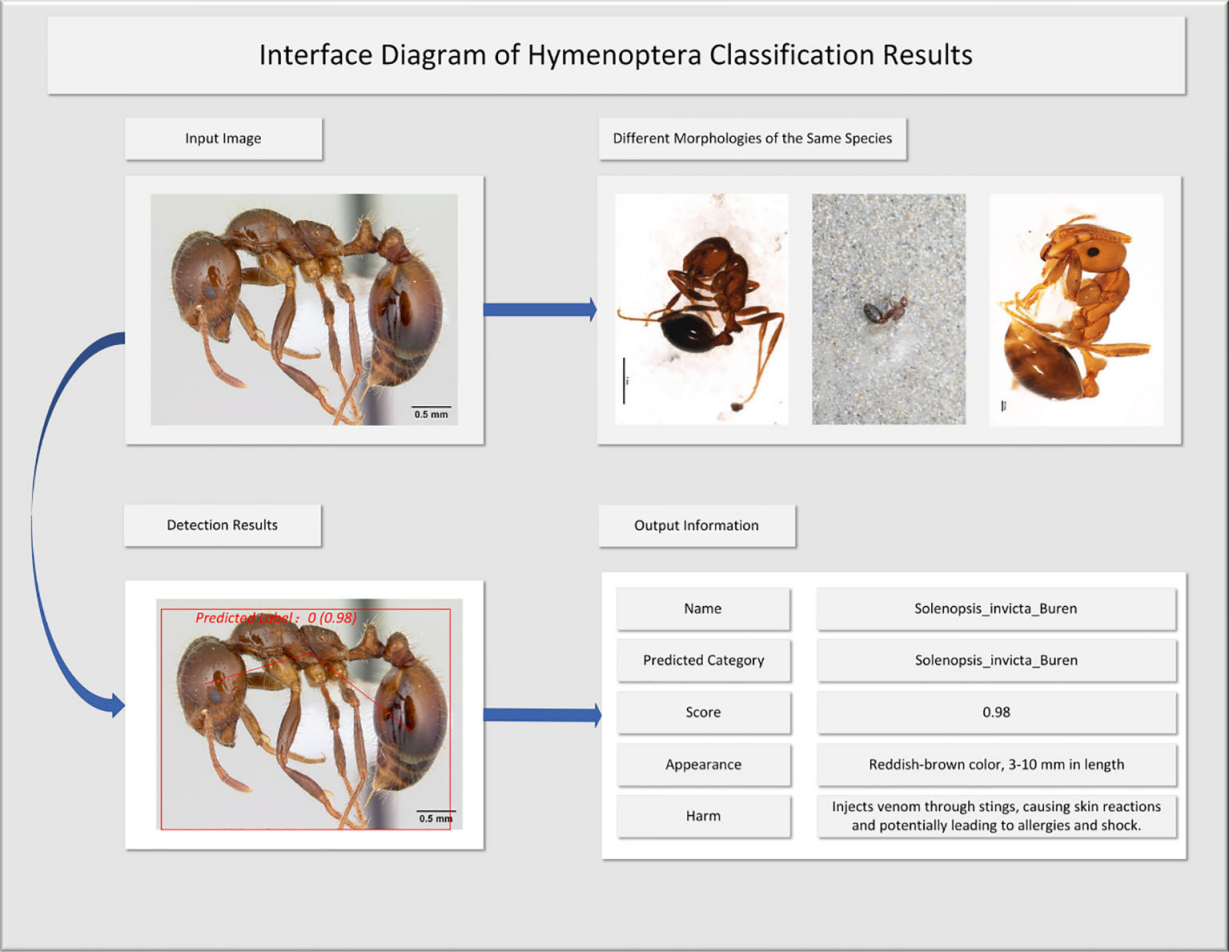
Diagram of insect detection and information analysis system.

An insect recognition system based on the model is designed, as shown in **Figure 6**. The system receives pest pictures inputted by users and automatically processes them to generate a detailed analysis report. The report consists of three parts: firstly, it displays pictures of different forms of pests to help users fully understand the diversity of their appearance characteristics; secondly, it generates a characteristic diagram of key parts of pests, labeling and analyzing the main structures (e.g., the head, thorax, abdomen), providing classification information and structural analysis; lastly, it outputs detailed information about the pests, including the name, detection category, classification confidence, description of their appearance, and potential hazard analysis.

This structured output makes the process of pest detection more intuitive and efficient, and allows users to quickly obtain information about the pests, understand the morphological characteristics and the degree of harm, which provides a strong support for the actual monitoring, prevention and control. At the same time, this structured information output method also provides researchers with convenient analysis tools, which helps to promote the promotion and deepening of pest detection technology in practical applications.

Based on the classification performance of our model on the Ant7C dataset, we found that there is still limitation when dealing with certain congeneric species. Such limitation lies mainly in the ability to discriminate subtle differences between key body regions (i.e., head, thorax, and abdomen). Imaging details in these regions are highly susceptible to external factors such as lighting, camera angle and complex backgrounds, posing a significant challenge to accurate recognition. At the same time, our practice of cutting images into too many small parts (e.g., separate tentacles, leg segments, or mouthparts) is undesirable. In complex contexts, small parts such as nodes and mouthparts are more susceptible to false detection or cropping bias, which in turn degrades the overall classification performance. Therefore, we finally chose the three levels of ‘head-thorax-abdomen’. This can adequately cover the key features while keeping the complexity of the detection and classification modules within acceptable limits. But such tasks need to discriminate subtle differences between key body regions (i.e., head, thorax, and abdomen). Imaging details in these regions are highly susceptible to external factors such as lighting, camera angle and complex backgrounds, posing a significant challenge to accurate recognition. Future work will also focus on exploring and integrating more advanced high-resolution feature extraction techniques with innovative data enhancement strategies. Our goal is to enhance the model’s ability to detect and recognize subtle differences between closely related species under challenging imaging conditions, thereby further improving classification accuracy.

## 4 Conclusion

In agricultural insect identification, inter-class similarity poses a significant challenge as certain insect species may exhibit highly similar appearances while demonstrating markedly different ecological impacts. For instance, Solenopsis invicta and Solenopsis geminata share striking visual resemblance, yet the former causes substantially greater damage to crops and ecosystems. Similarly, subtle morphological differences between Lasius_niger and Solenopsis invicta can lead to misidentification with serious consequences for insect management strategies.

To address the above challenges, we propose a model called DIRM. The model first extracts and crops key anatomical regions (head, thorax, and abdomen) using a DINO-based detector, and then processes these regions through an EfficientNet-B0 classification network enhanced with a global contextual enhancement module. Our experiments demonstrate that the preprocessing-enhanced cascade provides an 81.20% accuracy, and the complete DIRM reaches 95.48% after adding location-aware component position fusion, global contextual enhancement, and adaptive synergistic decision fusion. The adaptive synergistic mechanism proved to be the most effective, dynamically balancing the contribution of each anatomical region and improving both stability and accuracy. Visual analyses further validate the reliability of the model, demonstrating its pattern of attention in ant anatomy.

Although our proposed model has achieved excellent classification performance on the Ant7C dataset, its successful application to other insect datasets or a wider range of insect species is an important direction for future research and a key area for further improvement. Given the significant anatomical differences between different insect taxa, fine-tuning the model is crucial for practical applications (Li et al., 2023). Notably, our GCE framework and its adaptive synergistic mechanism have shown strong generalization potential, laying the groundwork for applying this core idea to other fine-grained image classification tasks. The limitation of the study also exists in the high-resolution images with more morphological details among the similar species dataset. We would focus on such expansion of our dataset and continue the evaluation of the cross-species generalization capability.

Subsequent research will focus on comprehensively evaluating and improving the model architecture’s cross-species portability and adaptability in different application scenarios to fully realize its practical value.

## Data Availability

The raw data supporting the conclusions of this article will be made available by the authors, without undue reservation.
